# Synthesis, Spectroscopic, Structural and Quantum Chemical Studies of a New Imine Oxime and Its Palladium(II) Complex: Hydrolysis Mechanism

**DOI:** 10.3390/molecules21010052

**Published:** 2016-01-21

**Authors:** Yunus Kaya, Veysel T. Yilmaz, Orhan Buyukgungor

**Affiliations:** 1 Department of Chemistry, Faculty of Arts and Sciences, Uludag University, Bursa 16059, Turkey; vtyilmaz@uludag.edu.tr; 2 Department of Physics, Faculty of Arts and Sciences, Ondokuz Mayis University, Samsun 55139, Turkey; orhanb@omu.edu.tr

**Keywords:** imine oxime, hydrolysis mechanism, DFT calculations, Pd(II) complex, crystal structure

## Abstract

In this work, we report synthesis, crystallographic, spectroscopic and quantum chemical studies of a new imine oxime, namely (4-nitro-phenyl)-(1-phenyl-ethylimino)-acetaldehyde oxime (nppeieoH). Spectroscopic and X-ray diffraction studies showed that nppeieoH is hydrolyzed in aqueous solution, forming nitroisonitrosoacetophenone (ninap) and the hydrolysis product binds to Pd(II) to yield [Pd(nppeieo)(ninap)]. The mechanism of the hydrolysis reaction has been theoretically investigated in detail, using density functional theory (DFT) with the B3LYP method. The vibrational and the electronic spectra of nppeieoH and its Pd(II) complex, the HOMO and LUMO analysis, Mulliken atomic charges and molecular electrostatic potential were also performed. The predicted nonlinear optical properties of both compounds are higher than those of urea.

## 1. Introduction

Molecules containing carbon-nitrogen double bonds are prevalent in both chemical and biological contexts. The two most common members of these molecules are oxime and imine oxime compounds. These compounds have quite common applications. They have been extensively used in analytical chemistry for the detection and separation of metal ions [1,2,3,4,5]. Moreover, some oximes and their complexes have been reported to have significant biochemical activity [6,7,8,9,10,11,12].

Concerning the role of the water molecule as a hydrolysis product in the oxime and imine oxime compounds, kinetic and mechanistic studies of these compounds are of special importance [13,14]. There have been few reports concerning their hydrolysis in the literature. The hydrolysis of benzophenone oxime was reported in 1934 [15]. Depuy and Ponder reported that the hydrolysis of various oximes in the presence of levulinic acid produced the corresponding carbonyl compounds [16]. The hydrolysis of *O*-(methylcarbamoyl)oximes in basic solutions was studied by Mrlina and Calmon [17]. In addition, the conversion of oximes into their parent carbonyl compounds was achieved by metal ion-assisted hydrolysis [18,19,20]. Moreover, the hydrolysis of cyclohexanone oxime has been theoretically proposed [21]. Recently, we studied the hydrolysis of an imine oxime, namely (1*E*,2*E*)-phenyl-[(1-phenylethyl)imino]-ethanal oxime (ppeieoH), in aqueous solution theoretically [14]. However, the theoretical study of the mechanism of the hydrolysis of nppeieoH has not been found in the literature.

Nowadays, on its merits, DFT is being applied as a computational method for calculating the structural properties of molecular systems; it provides greater accuracy in reproducing the experimental values of molecular geometry, vibrational frequencies, atomic charges, dipole moment, *etc.* [22,23,24]. Computational predictions of potential targets of bioactive small molecules have also received considerable interest during the last few decades. As part of this, docking is frequently used to predict the binding modes of small molecules to their targeted proteins; hence, it plays an important role in rational drug design [25,26]. Thus, computation provides a strong basis for experimental synthesis of new bioactive molecules and proposed pharmacophores.

In this study, we are reporting the synthesis and characterization of a new imine oxime (nppeieoH) and its palladium(II) complex. It was observed that nppeieoH hydrolyzed during the synthesis of the palladium(II) complex. In addition, using quantum mechanical methods, the hydrolysis mechanism was initially suggested as having two different pathways, which are before (**I**) and after (**II**) the complex is formed. Then, the activation energies of the two pathways of the conversion of imine oxime (nppeieoH) into carbonyl oxime (nitro-isonitrosoacetophenone (ninapH)) were compared to each other. The spectroscopic properties, such as IR and UV-Vis spectra, of both compounds are reported both experimentally and theoretically. The molecular electrostatic potential (MEP), Mulliken charges and first hyperpolarizability are also reported. In addition, this paper reports the single crystal X-ray structures of nppeieoH and [Pd(nppeieo)(ninap)].

## 2. Results and Discussion

### 2.1. Synthesis and Characterization

**Scheme 1:**
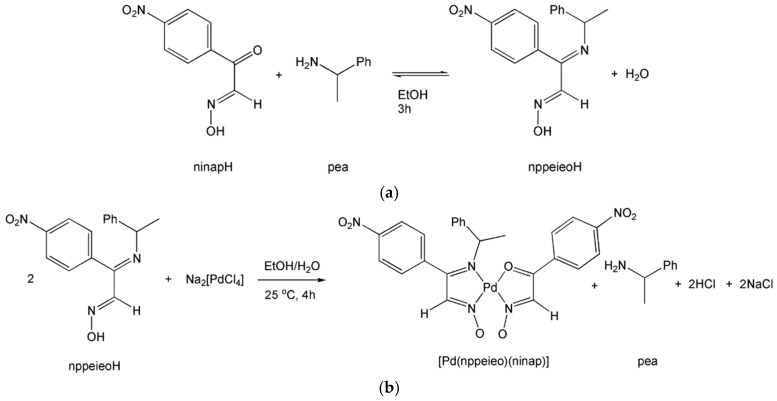
Syntheses of (**a**) (1*E*,2*E*)-nitrophenyl-[(1-phenylethyl)imino]-ethanal oxime (nppeieoH) and (**b**) [Pd(nppeieo)(ninap)].

A new imine oxime, (1*E*,2*E*)-nitrophenyl-[(1-phenylethyl)imino]-ethanal oxime (nppeieoH), is synthesized by the reaction of nitroisonitrosoacetophenone (ninapH) and 1-phenylethylamine (pea) in EtOH. [Pd(nppeieo)(ninap)] was obtained by the reaction of nppeieoH with Na_2_[PdCl_4_] in aqueous solution. nppeieoH and its palladium(II) complex were characterized by elemental analysis, IR, NMR, UV-Vis and X-ray diffraction analysis. X-ray diffraction analysis of the palladium(II) complex shows that the palladium(II) ion is coordinated in a distorted square-planar geometry by nppeieo and ninap, which is formed during the hydrolysis of nppeieo. The syntheses of nppeieoH and [Pd(nppeieo)(ninap)] are given in Scheme 1. nppeieoH and [Pd(nppeieo)(ninap)] were obtained in high yields, 88% and 82%, respectively. In addition, X-ray diffraction analyses of nppeieoH and its palladium(II) complex show that they are chiral and the crystal structures contain both enantiomers.

In nppeieoH, the CN bands were observed at 1605 and 1591 cm^−1^ as sharp bands, which are stretching vibrations of the imine and oxime groups, respectively. After coordination, these stretching vibrations were measured at 1601 and 1557 cm^−1^ as medium bands. The strong band at 1621 cm^−1^ is due to the absorption of the carbonyl group in the palladium(II) complex. The carbonyl group absorption was observed at 1640 cm^−1^ in the free ninapH. The stretching vibrations of imine and carbonyl groups at a relatively low frequency in the palladium(II) complex clearly indicate their participation in the coordination with the palladium(II) ion. The NO stretching, which appears in nppeieoH and ninapH at *ca*. 1000 cm^−1^, is observed at 1223 cm^−1^, and this is consistent with the previous findings [13]. In the ^1^H-NMR spectrum of the palladium(II) complex, the oxime OH proton does not appear, which was observed at δ = 12.30 ppm for the free nppeieoH. The signals of ^13^C-NMR are consistent with the structure of the palladium(II) complex. In the palladium(II) complex, the carbons of the C=O and three C=N groups resonate at *ca*. 176, 162, 144 and 142 ppm, respectively, while these chemical shifts of the carbon atoms of the carbonyl and imine groups are observed at *ca*. 180, 166, 144 and 141 ppm, respectively, in the free nppeieoH and ninapH ligands.

### 2.2. Hydrolysis Mechanism

**Figure 1:**
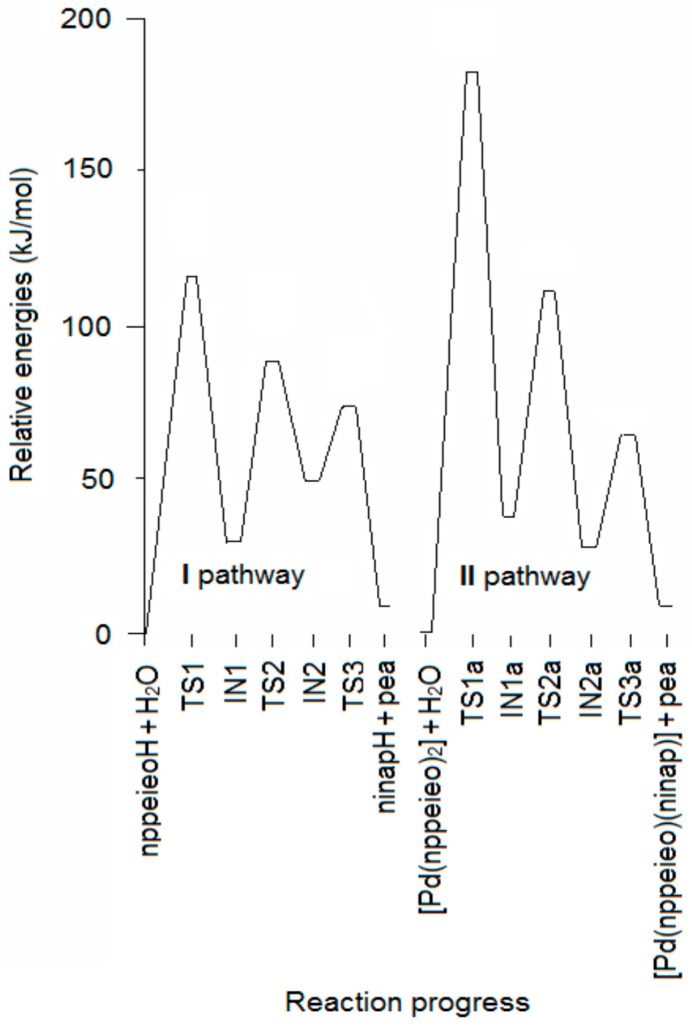
Energy profile for the hydrolysis of nppeieoH.

In this study, during the preparation of the palladium(II) complex of nppeieoH, we observed that the partial hydrolysis of the imine oxime ligand occurs during complexation in the presence of water, resulting in the corresponding carbonyl oxime (ninapH) and 1-phenyletanamime (pea). Two pathway mechanisms have been proposed for this process, which consists of hydrolyzed nppeieoH before (I) and after (II) the complex is formed in neutral aqueous solution. The two hydrolysis mechanisms subject to theoretical analysis are given in Scheme S1. In both pathways, the mechanism of the hydrolysis reaction of the nppeieoH molecule involves three steps, namely: (i) formation of a carbinolamine intermediate (imine oxime-IN1); (ii) transferring the hydrogen atom bonded oxygen atom to a nitrogen atom (Transition State 2 (TS2)-IN2); and (iii) dissociation of the carbinolamine to give the final carbonyl oxime (ninapH) and amine (pea) products (TS3-ninapH + pea). The corresponding potential energy surfaces (PESs) for the imine oxime hydrolysis for both pathways are shown in Figure 1, while the Gibbs free energies (Δ*G*) of the reactants, intermediates (IN), transition states (TS) and products are given in Table 1.

**Table 1:** Relative energy and negative frequency of the structures of the reaction paths for the hydrolysis of nppeieoH and [Pd(nppeieo)_2_]. TS, transition state; IN, intermediate; pea, 1-phenylethylamine.

Molecules	Relative Energy (kJ·mol^−1^)		Negative Frequency (cm^−1^)
	6-311G(d,p)	lanl2dz	
*nppeieoH*			
nppeieoH + H_2_O	0.0	0.0	-
TS1	109.9	112.3	−323
IN1	28.5	31.7	-
TS2	88.0	88.2	−1631
IN2	48.6	49.7	-
TS3	73.7	72.1	−294
ninapH + pea	10.7	13.8	-
*[Pd(nppeieo)_2_]*			
[Pd(nppeieo)_2_] + H_2_O		0.0	-
TS1a		177.4	−1361
IN1a		38.1	-
TS2a		108.7	−1574
IN2a		28.6	-
TS3a		58.2	−160
[Pd(nppeieo)(ninap)] + pea		12.5	-

**Figure 2:**
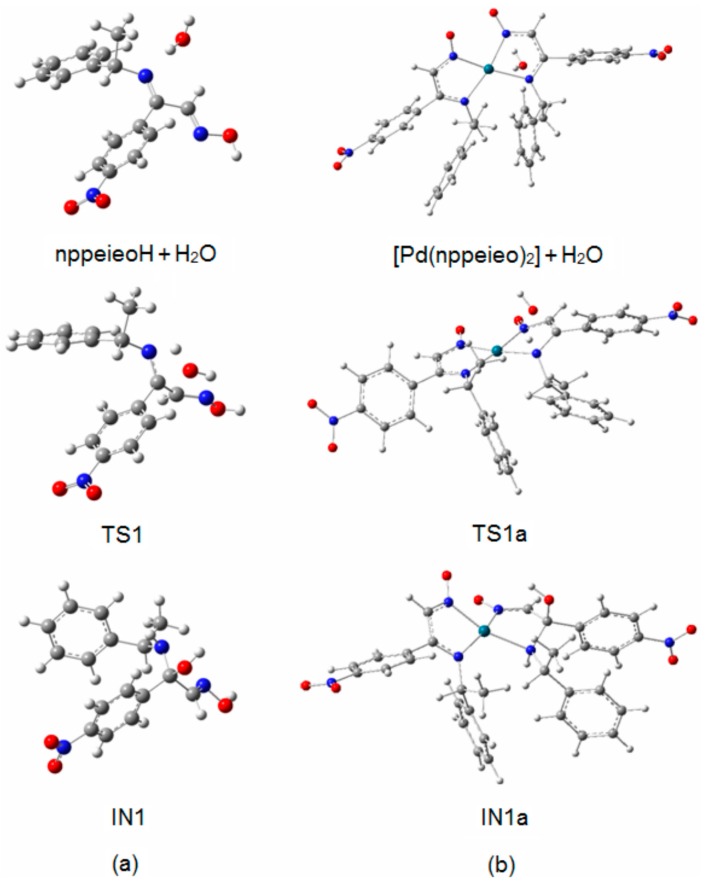
Optimized structures of imine oxime + H_2_O, TS1 and IN1: (**a**) nppeieoH; (**b**) [Pd(nppeieo)_2_].

Figure 2a,b shows the optimized structures in the first step, which are imine oxime + H_2_O, TS1 and IN1 for both pathways. The first step of the mechanism is the association of a single water molecule with the imine N atom of the nppeieoH molecule. One of the protons of the water molecule forms a hydrogen bond with the imine N atom of nppeieoH in nppeieoH + H_2_O and [Pd(nppeieoH)_2_] + H_2_O. The attack of the water molecule results in an interaction with the imine C atom through the O atom. The addition of water at that position consequently forms four-membered ring transition structures (TS1 and TS1a). The energy barriers (*E*_a_) of this step are quite high, being 112.3 and 177.4 kJ·mol^−1^, respectively, for the lanl2dz level. The activation energies for the I and II pathways related to TS1 and TS1a are shown in Figure 1, since the slow step is the first step in both pathways. These calculations show that the relative energy of TS1 is smaller than TS1a. Therefore, it was determined that the hydrolysis takes place before complex formation.

**Figure 3:**
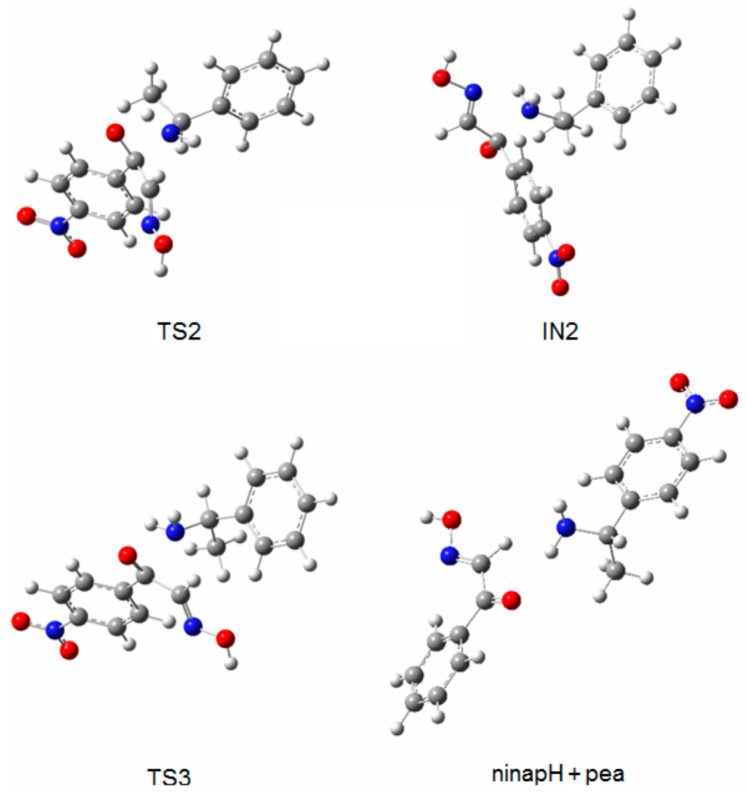
Optimized structures of TS2, IN2, TS3 and ninapH + pea.

**Figure 4:**
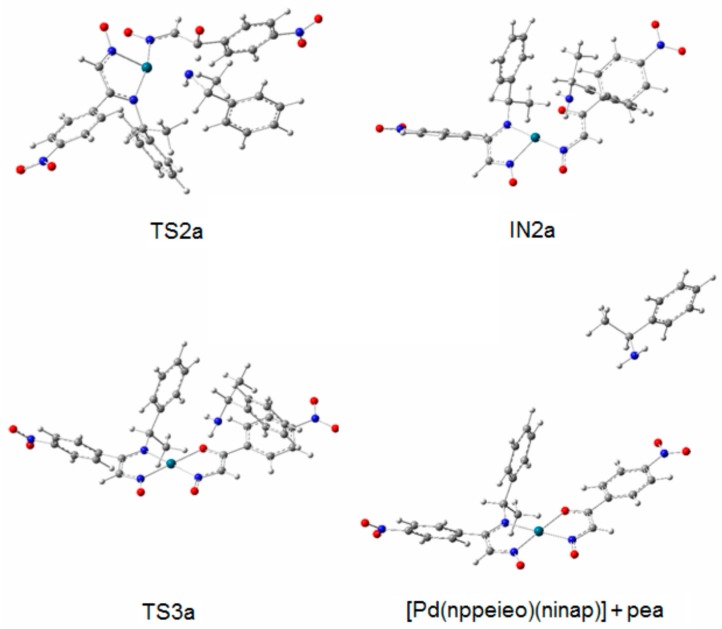
Optimized structures of TS2a, IN2a, TS3a and [Pd(nppeieo)(ninap)] + pea.

TS1 transforms into the tetrahedral intermediate (a carbinolamine, IN1). The barriers for the formation of the carbinolamine intermediate are rather low at 31.7 and 38.1 kJ·mol^−1^, respectively, for both pathways, compared to TS1 and TS1a. In the species from nppeieoH + H_2_O to IN1, the CN imine bond distance changes from *ca*. 1.25 to *ca*. 1.50 Å for both pathways, indicating the weakening of this bond. Subsequently, these are followed by the approach of the hydroxyl hydrogen to the amine nitrogen to form TS2 and TS2a with a relatively high energy barrier of *ca*. 88.0 kJ·mol^−1^. In the subsequent stages for both pathways (IN2, TS3 and ninapH + pea), the CN bond distances lengthen gradually from *ca*. 1.50–3.90 Å, leading to the cleavage of this bond to produce the corresponding carbonyl oxime (ninapH) and amine (pea) compounds. The optimized structures TS2, IN2, TS3 and ninapH + pea are given in Figure 3, while TS2a, IN2a, TS3a and [Pd(nppeieo)(ninap)] + pea are demonstrated in Figure 4. The Δ*G*_cal_ values of the formation of the final hydrolysis products for both pathways are 13.8 and 12.5 kJ·mol^−1^, respectively.

In fact, the hydrolysis of the title imine oxime is an equilibrium reaction with a calculated Δ*G*_cal_ value of *ca*. 13.0 kJ·mol^−1^ for both pathways. The presence of excess water molecules favors the formation of the products, as expected. As will be described below, this was observed experimentally during the synthesis of a palladium(II) complex of the imine oxime ligand.

### 2.3. Crystal Structure

**Figure 5:**
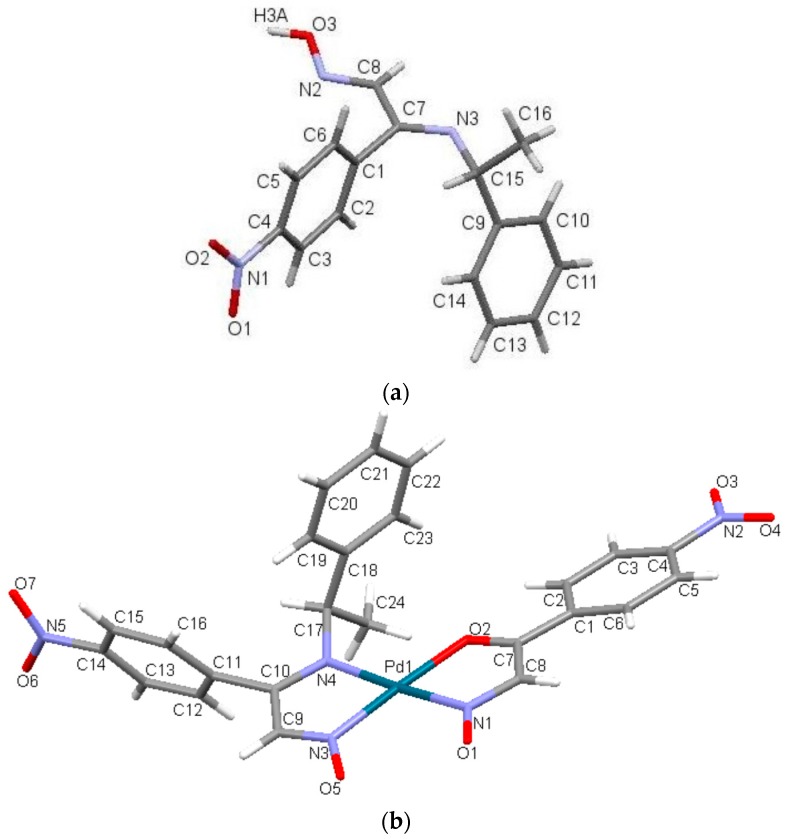
(**a**) Molecular view of nppeieoH. Selected bond lengths (Å) and (°): O3–H3a 0.913(3), N2–O3 1.391(16), C8–N2 1.267(2), C7–N3 1.281(2), C15–N3 1.486(2), C15–N3–C7 119.5(14), C8–N2–O3 112.0(13), N2–O3–H3a 104.8(15), O–H···N 1.904(2); symmetry code: x + 1/2, y + 1/2, z, O–H···O 1.834(3); symmetry code: 2 − x, y, 3/2 − z; (**b**) Molecular view of [Pd(nppeieo)(ninap)]. Selected bond lengths (Å) and (°): N1–O1 1.232(6), N3–O5 1.263(5), C9–N3 1.306(7), C10–N4 1.337(7), C17–N4 1.474(7), C8–N1 1.342(7), C7–O2 1.270(7), Pd1–N1 2.001(6), Pd1–N3 1.991(5), Pd1–N4 2.016(4), Pd1–O2 2.045(4), N1–Pd1–O2 80.4(2), O2–Pd1–N4 98.9(19), N3–Pd1–N4 80.7(2), N1–Pd1–N3 100.3(2), N3–Pd1–O2 176.2(2), N1–Pd1–N4 175.6(2).

The X-ray structures of nppeieoH and [Pd(nppeieo)(ninap)] are shown in Figure 5. In addition, the selected bond lengths and angles are seen in this figure caption. The title ligand and complex crystallize in a monoclinic space group C2/*c* with four and eight molecules in the unit cells, respectively. nppeieoH is not planar, and the overall molecular conformation can readily be defined in terms of the torsion angles. As expected, the imine oxime groups and nitrophenyl moiety are planar and almost perpendicular to each other with a dihedral angle of 88°. The bond distances in Figure 5 show that the double bonds in the molecule are localized between the C8 and N2 and the C7 and N3 atoms, resulting in bond lengths of 1.267 and 1.281 Å, respectively. The CN bond distances in nppeieoH are comparable to the corresponding bonds found in the similar oxime derivatives [13,14]. The crystal structure is formed by linking two molecules together with a water molecule. In addition, the molecules of nppeieoH and water molecules consist of two different intermolecular hydrogen bonds: first, the nppeieoH molecule linked by the O–H···O intermolecular hydrogen bond involving the hydroxyl H atom and the water O atom and the other intermolecular hydrogen bond between the water H atom and the imine N atom. These hydrogen bonds form a three-dimensional network (Figure S1).

As explained in Experimental Section, in the synthesis of the complex, the nppeieoH ligand was aimed at coordinating palladium(II). However, in the presence of water, some of the imine oxime ligands were hydrolyzed during the reaction, resulting in the products of a carbonyl oxime (ninap) and the corresponding amine (Scheme S1). Such imine bond (CN) hydrolysis is common in the reactions of imines and imine oximes [27,28,29,30,31,32,33,34,35]. Consequently, the nppeieo ligand together with the hydrolysis product (ninap) coordinate to palladium(II), forming a square-planar coordination geometry. The nppeieo and ninap ligands deprotonate to form the corresponding monoanions. nppeieo acts as a bidentate chelating ligand via two N atoms, while ninap behaves as a bidentate N, O donor. The Pd–O bond distance is measured as 2.045 Å. The Pd–N distances are 1.991 and 2.016 Å, being typical of those found in palladium(II) complexes of oximes [36,37,38,39,40,41,42,43,44,45,46,47]. The individual molecules of the complex were connected by weak C–H···O hydrogen bonds involving the [Pd(nppeieo)(ninap)] molecules to form a three-dimensional network (Figure S2).

### 2.4. Optimized Structure

The optimized parameters (bond lengths and bond angles) of nppeieoH and [Pd(nppeieo)(ninap)] obtained using the B3LYP/6-311++G(d,p) and lanl2dz basis sets are listed in Table S1, respectively. The optimized structures of nppeieoH and [Pd(nppeieo)(ninap)] are shown in Figure S3. The most important bonds of the imine oxime compounds are CN imine and oxime. These bond lengths of nppeieoH were calculated as 1.278 and 1.277 Å, respectively. On the other hand, these CN bond lengths were obtained as 1.330 and 1.352 Å in the palladium(II) complex. These results show that both CN bonds of the ligand weaken upon complexation. In addition, the carbonyl bond length was calculated as 1.312 Å in the [Pd(nppeieo)(ninap)] complex. The Pd–N bond distances of 2.032–2.081 Å are calculated, typical for the reported palladium(II) complexes containing imine oximes [13], while the Pd–O bond distance is 2.111 Å. The optimized parameters by DFT show a small difference from those obtained by X-ray diffraction, as seen Table S1 and Figure 5. The largest difference between the experimental observations and those obtained from the theoretical calculations is 0.051 Å in the O–H bond length.

### 2.5. Mulliken Atomic Charges

The Mulliken atomic charges for nppeieoH and its palladium(II) complex calculated at the B3LYP/6-311++G(d,p) and lanl2dz levels, respectively, in gas phase are presented in Figure S4. The Mulliken charge distribution of nppeieoH shows that the oxime oxygen atom is more negative (−0.760) as compared to azomethine nitrogen atoms (−0.590 and −0.005). The lower negative charges on N atoms are due to the charge transfer in O–H/N-type intra- or inter-molecular hydrogen bonds. The charges of the O atoms of the nitro group are −0.683 and −0.695, while the Mulliken charge of the N atom is calculated as 1.201 in the nitro group. On the other hand, in the palladium(II) complex, the charges of the all O and N atoms are negative, except the N atoms of nitro groups. However, these negative charges are lower than observed in nppeieoH. The palladium(II) atom has a positive charge, which is 0.380 in [Pd(nppeieo)(ninap)]. It has been noted that all hydrogen atoms are positively charged. It has also been observed that some C atoms are positive and some are negative. In nppeieoH, C2, C6, C10, C11, C13 and C14 are negatively charged atoms, while the remaining are positively charged. In the palladium(II) complex, C4, C7, C10, C11, C14 and C18 are positively charged atoms, while the others are negatively charged. Such a type of charge distribution generates the total dipole moment of 3.5517 Debye for nppeieoH and 1.8129 Debye for [Pd(nppeieo)(ninap)].

### 2.6. Frontier Molecular Orbitals

The HOMOs and LUMOs are known as Frontier molecular orbitals (FMOs), which played an important role for evaluating molecular chemical stability, chemical reactivity and the hardness/softness of the molecule [48]. The HOMO and LUMO energy, energy gap (ΔE), absolute electronegativity (χ), chemical hardness (η), softness (S) and electrophilicity index (ω) of nppeieoH and [Pd(nppeieo)(ninap)] are listed in Table 2 [49,50]. The HOMO acts as an electron donor, while the LUMO is an electron acceptor. The energy gap (ΔE) represents the chemical reactivity of compounds. For a system, a lower value of ΔE makes it more reactive or less stable. As depicted in Table 2, nppeieoH has a larger energy gap than its complex, [Pd(nppeieo)(ninap)]. The energy gap, ΔE, is directly involved in the hardness/softness of a chemical species. A higher value of ΔE represents more hardness or less softness of a compound; thus nppeieoH is referred to as a hard molecule when matched with its complex [51]. The global reactivity descriptor chemical potential (I), which is represented by HOMO energy, occurs from the charge distribution between two systems having different chemical potentials. Here, both compounds act as electrophiles, and hence, their electronic potentials (I) are negative. Another global reactivity descriptor electrophilicity index (ω) describes the electron accepting ability of the systems quite similar to η and I. High values of the electrophilicity index increase the electron accepting abilities of the molecules. Thus, the electron accepting abilities of nppeieoH and its complex are arranged in the following order: [Pd(nppeieo)(ninap)] > nppeieoH.

**Table 2:** HOMO and LUMO energies, energy gap (ΔE), absolute electronegativity (χ) chemical hardness (η), softness (S) and electrophilicity index (ω) of nppeieoH and [Pd(nppeieo)(ninap)].

Compound Global Reactivity Descriptors	nppeieoH	[Pd(nppeieo)(ninap)]
E (HOMO, a.u.)	−0.259	−0.246
E (LUMO, a.u.)	−0.110	−0.146
ΔE (eV)	4.053	2.720
χ	−5.018	−5.372
η	2.026	1.360
S	0.247	0.368
ω	6.214	10.610

**Figure 6:**
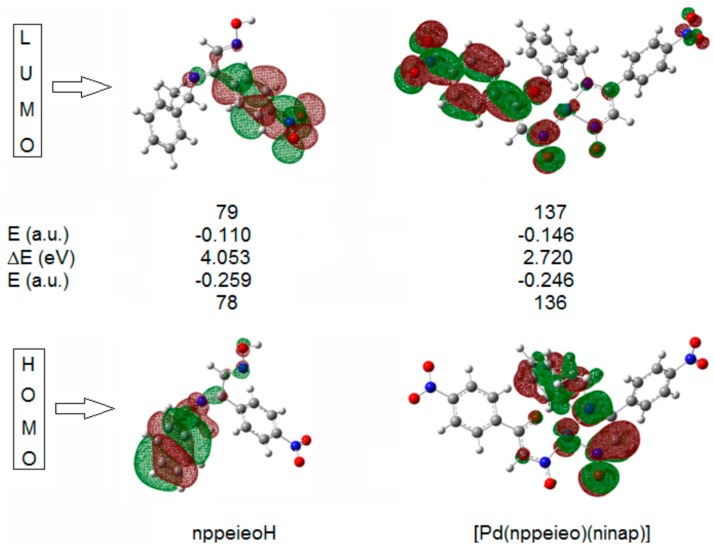
Frontier molecular orbitals of nppeieoH and its palladium(II) complex.

In the HOMO of both compounds, the electron density mainly delocalized over the associated phenyl ring, while in the LUMO orbital, this density is delocalized on the imine oxime group, as shown in Figure 6.

### 2.7. Molecular Electrostatic Potential Map

**Figure 7:**
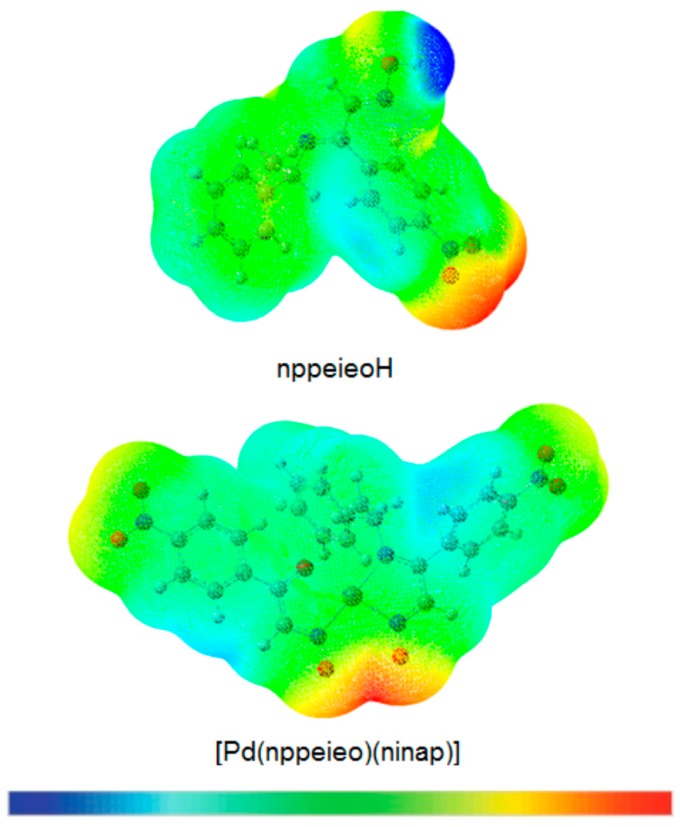
Molecular electrostatic potential (MEP) plot of nppeieoH and its palladium(II) complex.

The chemical reactivity of the compounds is easily determined with the help of the molecular electrostatic potential map (MEP), which differentiates the electrophilic and nucleophilic sites in a molecule quite easily [52]. For this purpose, the MEPs have been calculated for nppeieoH and [Pd(nppeieo)(ninap)] at the B3LYP/6-311++G(d,p) and lanl2dz levels, respectively. In the MEP plots, as represented in Figure 7, the negative regions represented by the red color are the preferable sites for electrophilic attack, and the positive regions represented by the blue color are favored for nucleophilic attack. Here, the negative potentials are generated over the electronegative oxime O, azomethine N and nitro O atoms, whereas the H-atoms have a positive potential region in the structures. These negative and positive sites help to predict the regions in a compound responsible for non-covalent interactions [53].

### 2.8. Vibrational Spectroscopy

**Figure 8:**
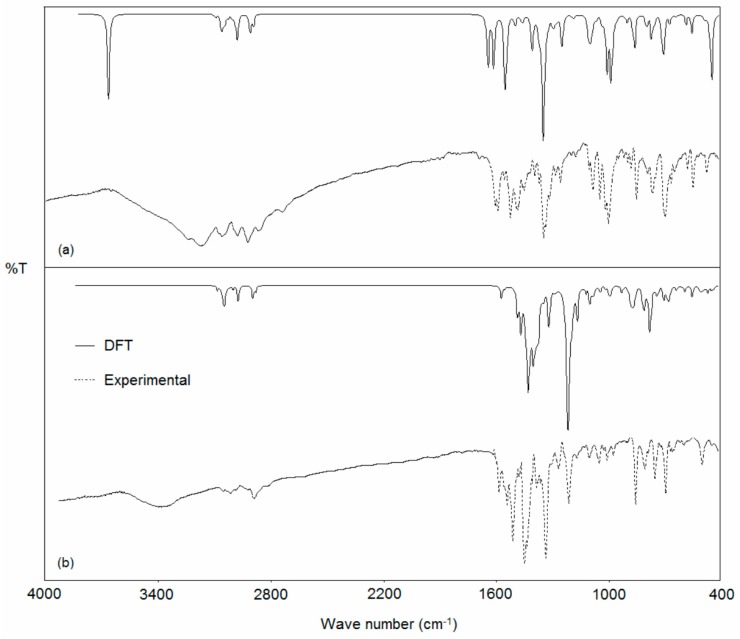
Experimental and calculated IR spectra of the (**a**) nppeieoH and (**b**) [Pd(nppeieo)(ninap)].

The harmonic vibrational frequencies for nppeieoH were calculated by using the DFT method with the 6-311++G(d,p) basis set, while [Pd(nppeieo)(ninap)] was calculated by using the lanl2dz basis set. The corresponding frequencies along with the assignments and intensities are given in Table 3, while the observed and calculated vibrational spectra are given in Figure 8. The calculated frequencies with an intensity less than 10 were not taken into consideration. It can be seen that the experiment has a better correlation with the calculations. In the IR spectra of nppeieoH, the O–H stretching vibration of the oxime was calculated at 3659 cm^−1^, while observed at 3258 cm^−1^ [54,55,56]. The deviation between the experimental and calculated values seems to be significant for the hydroxyl group frequencies with a difference of 401 cm^−1^. Due to the nature of this vibration mode, its frequency is very sensitive to the crystalline state, in which the hydrogen bonding interactions involving this group are present, as discussed above, and, thus, exhibits a much larger deviation from the calculated values. At the same time, in the high wavenumber region of the spectra, the anharmonicity can explain substantial differences between the experimental and calculated values [57]. This stretching of the oxime group was not observed in the palladium(II) complex, due to the fact that the imine oxime loses this hydroxyl proton upon complexation. Significant vibration bands of the ligands and their metal complexes may be used for determining the ligands’ mode of coordination by the comparative analysis of the spectra of the ligand and the complex, in particular in relation to the changes observed after complexation. The experimental CN bands in nppeieoH were observed as sharp bands at 1604 and 1597 cm^−1^, which were computed at 1647 and 1634 cm^−1^, respectively [54,58,59]. These absorption bands were observed at 1601 (calcd. 1587 cm^−1^) and 1562 (1499 cm^−1^) cm^−1^, respectively in the palladium(II) complex. In addition, strong characteristic absorption due to NO stretching vibration is observed at 999 cm^−1^ and calculated at 1000 cm^−1^, which consists of 62% NO stretching and 16% bending of CH_aliph._ [54,55,56,58]. A substantial change is also observed in the NO stretching; the NO absorptions occurred at 1218 cm^−1^ (calcd. 1222 cm^−1^) for [Pd(nppeieo)(ninap)], indicating an increase in the double bond character of the NO bond upon complexation [27,36]. Vibrational modes in the low wave number region of the spectra contain ν(M–O) and ν(M–N) stretching together with the contributions of several other modes. [Pd(nppeieo)(ninap)] shows two bands at 575 cm^−1^, which can be attributed to mixed ν(M–O) and ν(M–N) (calcd. 549 cm^−1^) and 260 cm^−1^, which can be attributed to ν(M–N) (calcd. 253 cm^−1^) [13,14].

**Table 3:** Experimental and calculated FT-IR spectra for nppeieoH and its palladium(II) complex together with their assignment ^a^ (wavenumber in cm^−1^).

nppeieoH				[Pd(nppeieo)(ninap)]			
Assignments (PED > 10)	Exp.	IR	*I*	Assignments (PED > 10)	Exp.	IR	*I*
νOH (97)	3258br	3659	158	νCH_phen._ (92)	3108w	3097	31
νCH_phen._ (95)	3078w	3052	20	νCH_phen._ (97)	3073w	3090	26
νCH_phen._ (91)		3041	19	νCH_phen._ (89)	3051w	3050	10
νCH_methyl_ (88)	2983w	2982	15	νCH_phen._ (95)		3020	37
νCH_methyl_ (90)	2931w	2970	32	νCH_methyl_ (89)	2943w	2942	28
νCH_methyl_ (87)		2904	24	νCH_HCNO_ (98)		2927	21
νCH_HCNO_ (99)	2876w	2887	23	νCO (37) + νCN (27) + νCC_phen._ (24)	1601m	1587	24
νCN_imine_ (67) + νCN_oxime_ (21)	1604s	1647	11	νCN (57) + νCC_phen_ (12)	1562m	1499	61
νCN_oxime_ (61) + νCN_imine_ (18)	1597s	1633	76	νCC_phen._ (49)	1532s	1482	129
νCC_phen._ (54) + ™CH_phen._ (21)		1605	58	™CH_phen._ (63)		1474	13
νCC_phen_ (48) + ™CH_phen._ (34)		1604	15	™CH_phen._ (71) + νCN (17)	1468s	1464	32
νCC_phen._ (50) + ™CH_phen._ (18)	1563w	1600	16	™CH_phen._ (41) + ™CH_aliph._ (23)	1451s	1446	351
νNO_NO2_ (51) + νCC_phen._ (17)	1521s	1541	207	™CH_aliph._ (33) + νCN (21)		1436	341
™CH_phen._ (71)	1493s	1490	13	™CH_phen._ (64)		1419	26
™OH (47) + ™CH_phen._ (33)	1403w	1400	36	™CH_phen._ (64) + νCO (13) + νCN (10)	1399m	1416	261
™OH (43) + ™CH_phen._ (39)		1395	29	™CH_phen._ (38) + ™CH_aliph._ (16)		1403	123
™CH_HCN_ (46) + ™CH_phen._ (19)		1356	25	νCN_NO2_ (68) + νCN (17)	1353s	1389	222
νCN_NO2_ (91)	1349s	1340	373	™CH_aliph._ (53)		1382	21
™CH_phen._ (38) + ™CH_aliph._ (26)	1264m	1282	13	™CH_phen._ (48) + νCN_NO2_ (18)		1371	18
™OH (28) + ™CH_HCN_ (25)	1201w	1239	62	™CH_phen._ (49)		1355	21
νC–N (37) + νC–C (20)	1092m	1095	26	™CH_aliph._ (38)		1333	23
νC–N (18) + νC–C(32)		1088	34	™CH_aliph._ (41) + νCC_phen._ (12)	1389w	1329	93
™CH_aliph._ (36)		1073	20	™CH_phen._ (27) + ™CH_aliph._ (14)		1319	20
νNO(62) + ™CH_aliph._ (16)	999s	1000	78	™CH_aliph._ (43)		1234	30
νNO(32) + ™CC_phen._ (28)		994	33	νCN_NO2_ (63) + νNO (14)		1227	314
νNO(24) + ™CH_aliph_. (46)		976	113	νNO (64) + νCN_NO2_ (23)	1218m	1222	723
™CH_aliph._ (27)	887w	893	12	™CH_aliph._ (33) + ™CH_phen._ (24)		1218	25
™NO_NO2_ (48) + ™CC_phen._ (27)	856m	853	50	™CH_phen._ (46) + νCN (16)	1180w	1202	79
γCC_phen._ (47) + γCH_aliph_. (27)	771m	787	25	™CH_phen_. (48)		1174	31
γCC_phen_ (52)		762	28	™CH_phen_. (54)		1173	46
γCC_phen._ (35) + γNO_NO2_ (27)	702s	698	63	™CH_phen._ (26) + ™CH_aliph._ (14)	1112w	1104	34
γCC_phen_ (63)		697	30	™CH_phen_ (43)		1099	13
γCN(37) + γCC_phen_ (25)	652w	662	14	νC–N (47) + νC–N (12)	1049w	1083	13
γCC_phen._ (42)	555m	546	26	™CC_phen._ (58) + ™CH_phen._ (10)	984w	995	25
γOH (61)	486w	442	125	νC–N (26) + ™CH_aliph_. (18)		933	14
				γCC_phen_ (73)		887	36
				γCC_phen_. (68)	856m	871	72
				™CH_aliph._ (47)	810w	818	38
				™CH_aliph._ (32)		808	23
				™CH_aliph._ (23)		804	36
				™NO_NO2_ (38) + ™CC_phen._ (22)		780	66
				™NO_NO2_ (41) + γCC_phen._ (27)	756m	774	103
				γCC_phen._ (38)		736	29
				γCC_phen._ (56)	698m	699	42
				γCC_phen._ (32) + γNO_NO2_ (37)		678	30
				γCC_phen._ (28) + γNO_NO2_ (21)	671w	677	27
				νPdO (27) + νPdN (12) + δONC (12)	575vw	549	23
				γCNCC (38) + γCC_phen._ (22)	488w	465	10
				γCNCC (28) + γCC_phen._ (21)	451vw	462	10
				γCC_phen._ (37)		429	10
				νPdN (14), γCC_phen._ (14), γCNCC (10)	260w	253	20
				νPdN (21), δPdCN (15)		244	18

^a^ br: broad, s: strong, m: medium, w: weak, vw: very weak; ν: stretching, δ: in-plane bending, γ: out-of-plane bending, τ: twist, phen. = phenyl, aliph. = aliphatic, Exp. = Experimental, PED = Potential Energy Distribution; scaled factor: 0.958 in the range of 4000–1700 cm^−1^; 0.978 in the range of 1700–400 cm^−1^ for nppeieoH and 0.9614 for [Pd(nppeieo)(ninapH)].

### 2.9. Electronic Absorption Spectra

**Figure 9:**
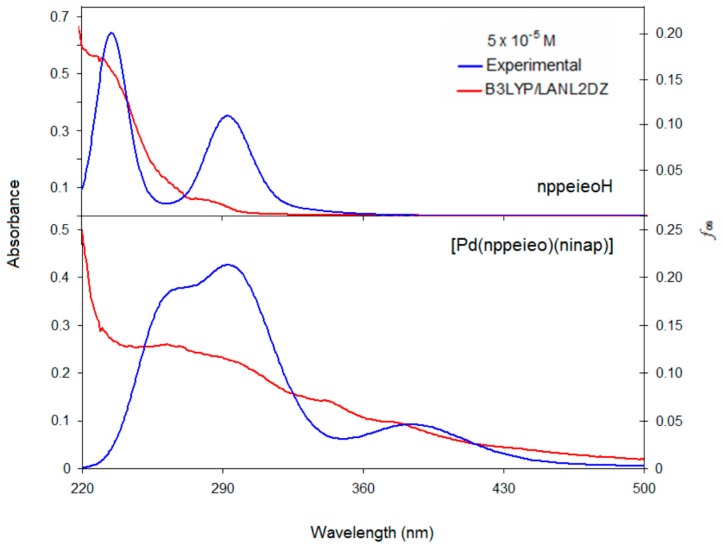
Experimental and calculated electronic spectra of nppeieoH and its palladium(II) complex.

The UV-Vis absorption spectra of nppeieoH and its complex were measured in EtOH. The absorption bands of the compounds were assigned based on time-dependent (TD)-DFT. The calculated excited states, absorption bands, oscillator strengths (*f_os_*), transition configuration and their assignments are given in Table 4, while the absorption spectra of these compounds are presented in Figure 9. The assignment of the calculated transitions to the experimental bands is based on the criterion of the energy and oscillator strength of the calculated transitions. The absorption bands of nppeieoH appear at around 231 and 282 nm. The absorption band at 231 nm can be mainly assigned to a superposition of three calculated bands between 233 and 242 nm. We ascribe the absorption band at 282 nm to the calculated transition at 293 nm with oscillator strength of 0.3334. Both transitions can be ascribed to the π→π* transition.

**Table 4:** Experimental and calculated electronic transitions, oscillator strengths and their assignments for nppeieoH and [Pd(nppeieo)(ninap)] ^a^.

Exp. (nm)	ε	Calcd. (nm)	*f_os_*	Major Contribution (CI Coeff.)	Character
NppeieoH					
282	0.1116	293	0.3334	H–5 → L (87%)	π (phen.) → π* (imineoxime)
		282	0.0300	H → L + 1 (84%)	π (phen.) → π* (phen.)
		242	0.0533	H–4 → L + 1 (57%)	π (phen.) → π* (phen.)
				H–5 → L + 1 (25%)	
		236	0.2036	H–6 → L + 1 (38%)	π (oxime) → π* (imineoxime)
231	1.0866	233	0.3864	H–6 → L + 1 (22%)	π (phen.) → π* (phen.)
				H–5 → L + 1 (13%)	π (imineoxime) → π* (phen.)
[Pd(nppeieo)(ninap)]					
376	0.1904	415	0.1391	H-4 → L (74%)	d(Pd)/π (imineoxime)→π* (imineoxime)
341	0.2839	346	0.2152	H-7 → L (30%)	π (imineoxime) → π* (imineoxime)
		329	0.0495	H–8 → L (32%)	π (phen.) → π* (imineoxime)
		324	0.1328	H–5 → L + 2 (33%)	π (phen.) → d(Pd)/π* (imineoxime)
295	0.4526	323	0.2073	H–7 → L (18%)	π (oxime) → π* (imineoxime)
				H–8 → L (13%)	
		296	0.1196	H–7 → L + 1 (27%)	π (imineoxime) → π* (phen.)
		289	0.1748	H–7 → L + 1 (23%)	π (imineoxime) → π* (phen.)
261	0.5164	287	0.2047	H−10 → L + 1 (39%)	π (phen.) → π* (phen.)

^a^ ε = molar absorption coefficient (×10^4^, dm^3^·mol^−1^·cm^−1^), *f_os_* = oscillator strength, H = highest occupied molecular orbital, L = lowest unoccupied molecular orbital, phen. = phenyl.

The absorption bands of the title complex appear at around 261, 295, 341 and 376 nm (Figure 9). To understand the transition processes, the calculated absorption transition diagram is shown in Figure S5. The high energy absorption at 261 nm is contributed by the electron excitation from HOMO−10 to LUMO + 1 at 287 nm with oscillator strength of 0.2047. The HOMO−10 and LUMO + 1 orbitals are localized on the phenyl ring, so this absorption can be ascribed to a π (phenyl (phen.)) → π* (phen.) transition. The absorptions at 295 and 341 nm are contributed by electron excitation from HOMO–7 to LUMO and HOMO–8 to LUMO. These orbitals have dominantly a π character, and thus, the absorption at 295 and 341 nm can be attributed to a π → π* transition (Figure S5). The low energy transition at 376 nm (calcd. 415 nm) originates from the electron transition between HOMO–4 and LUMO. The HOMO–4 orbital is composed of 38% d(Pd) and 55% p(imineoxime), whereas the LUMO is dominantly localized on the imine oxime group (68%). Therefore, the absorption may be assigned mainly to an ligand to ligand charge transfer (LLCT) transition, including metal to ligand charge transfer MLCT transition.

### 2.10. Non-Linear Optical Properties

A good non-linear optical (NLO) material has been frequently used in communication technology, signal processing, optical switches and optical memory devices. The non-linear optical properties originate with delocalized π electrons of a compound and increase with increasing conjugation in the compound. The presence of an electron donor group and an electron acceptor group also enhances the non-linear optical properties. The total static dipole moment (μ), the linear polarizability (α) and the first hyperpolarizability (β) using the x, y, z components are calculated using the following equations [60]:

(1) $$\mu =\sqrt{\mu_{x}^{2}+\mu_{y}^{2}+\mu_{z}^{2}}$$

(2) $$\alpha =\frac{\alpha_{xx}+\alpha_{yy}+\alpha_{zz}}{3}$$

(3) $$\beta =\sqrt{{\left(\beta_{\mathrm{xxx}}+\beta_{\mathrm{xyy}}+\beta_{\mathrm{xzz}}\right)}^{2}+{\left(\beta_{\mathrm{yyy}}+\beta_{\mathrm{xxy}}+\beta_{\mathrm{yzz}}\right)}^{2}+{\left(\beta_{\mathrm{zzz}}+\beta_{\mathrm{xxz}}+\beta_{\mathrm{yyz}}\right)}^{2}}$$

The dipole moment (μ), the linear polarizability (α) and the first hyperpolarizability (β) were calculated at the B3LYP/6-311++G(d,p) level for nppeieoH and lanl2dz basis set for [Pd(nppeieo)(ninap)]. The value of the first hyperpolarizability is determined in atomic units (a.u.) and then converted to electrostatic units (e.s.u.) using the conversion factor 1 a.u. = 8.6393 × 10^−33^ cm^5^·e.s.u.^−1^. The calculated dipole moment (μ), linear polarizability (α) and the first hyperpolarizability (β) in nppeieoH are 3.5517 D, 34.484 Å^3^ and 7.938 × 10^−30^ cm^5^·e.s.u.^−1^, respectively, while μ, α and β of [Pd(nppeieo)(ninap)] were calculated as 4.8129 D, 58.904 Å^3^ and 19.354 × 10^−30^ cm^5^·e.s.u.^−1^, respectively. Urea is one of the reference materials and frequently used for comparative purpose in the study of the NLO properties. The calculated values of μ, α and β for the selected compounds are greater than those of urea (the μ, α and β of urea are 1.3732 D, 3.8312 Å^3^ and 0.37289 × 10^−30^ cm^5^·e.s.u.^−1^ obtained by the B3LYP/6-311++G(d,p) method). Theoretically, the first-order hyperpolarizability (β) of [Pd(nppeieo)(ninap)] is 51.9-times higher than urea. These results indicate that the selected compounds possess good non-linear optical properties [48,61].

## 3. Experimental Section

### 3.1. General

The elemental analyses (C, H and N) were performed using a EuroEA 3000 CHNS elemental analyzer (Eurovector, Milano, Italy). UV-Vis spectra were measured on a Lambda 35 UV/Vis spectrophotometer (Perkin-Elmer, Waltham, MA, USA) using 1 × 10^−4^ M DMSO solution in the 200–800 nm range. IR spectra were recorded on a Nicolet 6700 FT-IR spectrophotometer (TurkeyThermo, Madison, WI, USA) as KBr (in the frequency range 4000–400 cm^−1^) and CsI (in the frequency range 400–250 cm^−1^) pellets. ^1^H-NMR (400 MHz) and ^13^C-NMR (100 MHz) spectra were recorded on a Mercury plus spectrometer (Salt Lake City, UT, USA) in DMSO-*d*_6_, and TMS was used as an internal standard.

### 3.2. Synthesis of nppeieoH and Its Palladium(II) Complex

1-Phenylethylamine (0.61 g, 5 mmol) dissolved in 5 mL EtOH was added dropwise in a 10-mL EtOH solution of nitroisonitrosoacetophenone (0.97 g, 5 mmol), and the resulting solution was stirred at room temperature for 3 h. Well-shaped prisms of nppeieoH.1/2H_2_O were obtained by slow evaporation at room temperature within 3 days. Yield 88%. M.p. 132.8–133.0 °C. Anal. calcd. for C_16_H_15_N_3_O_3_ (297.3 g·mol^−1^): C, 64.64; H, 5.09; N, 14.13. Found: C, 64.51; H, 5.13; N, 14.05%. ^1^H-NMR (400 MHz, DMSO-*d*_6_, 298 K, δ*/*ppm): 12.30 (s, 1H, H–ONC); 8.09 (s, 1H, H–CNO); 7.67–7.42 (m, 9H); 4.51 (m, 1H, CH); 2.32 (d, 3H, CH_3_). ^13^C-NMR (100 MHz, DMSO-*d*_6_, 298 K, δ/ppm): 166.08, 144.13 (C=N); 136.44–126.41; (C-phenyl), 59.87 (CH), 21.48 (CH_3_). (Solid KBr pellet): ν(cm^−1^) 3173br, 3063m, 3059m, 2975m, 2920m, 1605s, 1591s, 15244s, 1485m, 1349s, 1260w, 1085m, 1048m, 1002s, 856m, 770m, 701s, 553m, 483w.

A solution of nppeieoH.1/2H_2_O (0.306 g, 1 mmol) in ethanol (30 mL) was added drop-wise with stirring to a 10-mL aqueous solution of Na_2_[PdCl_4_] (0.147 g, 0.5 mmol), and then, this solution was stirred for 4 h at room temperature. The volume of the solutions was reduced to 10–15 mL under vacuum, and then, the orange-red precipitate was filtered off and recrystallization from DMSO, yielding orange single crystals. Yield 82%. M.p. 195–199 °C (decomposition). Anal. calcd. for C_24_H_19_N_5_O_7_Pd (595.9 g·mol^−1^): C, 48.38; H, 3.21; N, 11.75. Found: C, 48.21; H, 3.22; N, 11.64%. ^1^H NMR (400 MHz, DMSO-*d*_6_): δ 8.01, 8.24 (s, 2H, H–CNO), 7.75–7.21 (m, 13H, H-phenyl), 4.58 (m, 1H, CH), 2.03 (d, 3H, CH_3_). ^13^C-NMR (100 MHz, DMSO-*d*_6_): δ 176.04 (C=O), 162.71, 144.03, 142.23 (C=N), 135.68–125.96 (C-phenyl), 60.21 (CH), 20.52 (CH_3_). (Solid KBr pellet): ν(cm^−1^) 3105w, 3065m, 3035w, 2962w, 2934m, 1601m, 1557m, 1526s, 1464s, 1346s, 1223m, 1109w, 1056w, 1046w, 1014w, 980w, 857s, 806w, 754m, 696m, 497w, 343w, 254w. UV-Vis in 1 × 10^−5^ M DMSO solution, λ_max_/nm (ε/dm^3^·mol^−1^·cm^−1^): 429 (8380), 348 (9190), 285 (23470).

### 3.3. X-ray Crystallography

The intensity data of nppeieoH and its palladium(II) complex were collected using a IPDS II diffractometer (STOE, Darmstadt, Germany) with graphite-monochromated MoK_α_ radiation (λ = 0.71073 Å). The structures were solved by direct methods and refined on *F*^2^ with the SHELX-97 program [62]. All non-hydrogen atoms were found from the difference Fourier map and refined anisotropically. All hydrogen atoms were positioned geometrically and refined by a riding model. The details of data collection, refinement and crystallographic data are summarized in Table S2.

### 3.4. Theoretical Calculations

In the present work, the Becke-Lee-Yang-Parr functional (B3LYP) method [63] was adopted, and all calculations were performed using the GAUSSIAN 03 program package [64]. Calculations of neutral hydrolysis mechanism of nppeieoH were performed using the 6-311G(d,p) and lanl2dz basis sets, while [Pd(nppeieo)(ninap)] was calculated at the lanl2dz level. Harmonic frequencies of the structures were calculated at the same method and basis sets to find a local minima (all positive force constants) or transition states (one imaginary force constant only). General solvent/environment effects were modelled using the integral equation formalism variant of the polarizable continuum model (IEFPCM) [65,66,67] with a dielectric constant (ε) of 24.85, *i.e.*, ethanol as the bulk solvent. Specifically, single point calculations were performed at the IEFPCM (ε = 24.85)-B3LYP/6-311G(d,p) level of theory based on the above optimized geometries. The free energies of the systems in aqueous continuum were calculated by adding the thermal correction for the Gibbs free energy obtained from frequency calculations of the systems in the gas phase, to the energies in bulk solvent.

Geometry optimizations of nppeieoH and its palladium(II) complex were started from the X-ray experimental atomic position and fully optimized at the B3LYP and 6-311++G(d,p) level for nppeieoH and the lanl2dz level for the complex. For all of the spectroscopic and the physicochemical calculations in this study, optimized structural parameters were used. The harmonic vibrational frequency obtained calculations were scaled by 0.958 [68] for the 4000–1700 cm^−1^ and 0.978 [69] for the 1700–400 cm^−1^ ranges, respectively, for nppeieoH, and 0.9614 [70], for [Pd(nppeieo)(ninap)]. Vibrational band assignments were made using the GaussView molecular visualization program [71]. Furthermore, theoretical vibrational spectra of the compounds were interpreted by means of Potential Energy Distributions (PEDs) using the VEDA 4 program [72]. The electronic absorption spectra were calculated using TD-DFT in EtOH for both molecules using IEFPCM. The orbital contribution was analyzed using the GaussSum software [73]. In addition, optimized structural parameters have been used in evaluating frontier molecular orbitals, Mulliken charges, molecular electrostatic potential maps (MEP) and linear first hyperpolarizability properties.

The appendix is an optional section that can contain details and data supplemental to the main text. For example, explanations of experimental details that would disrupt the flow of the main text, but nonetheless remain crucial to understanding and reproducing the research shown; figures of replicates for experiments of which representative data is shown in the main text can be added here if brief, or as Supplementary Materials. Mathematical proofs of results not central to the paper can be added as an appendix.

## 4. Conclusions

In this work, a new imine oxime, namely (4-nitro-phenyl)-(1-phenyl-ethylimino)-acetaldehyde oxime (nppeieoH) and its palladium(II) complex, [Pd(nppeieo)(ninap)], have been synthesized and characterized by various techniques, including IR, NMR, UV-Vis, elemental analysis and X-ray single crystal determination. The X-ray single crystal analysis of [Pd(nppeieo)(ninap)] shows that the nppeieoH molecule is hydrolyzed, and the hydrolysis product is joined in the complex. Therefore, the mechanism of an imine oxime (nppeieoH) in neutral aqueous solution was studied in detail in a solvent environment, using the IEFPCM continuum model. Theoretically, two pathway mechanisms were proposed for this process, which consists of hydrolyzing of nppeieoH before the complex is formed (I) and hydrolyzing of [Pd(nppeieo)_2_] after the complex is formed (II). These two hydrolysis mechanisms were studied with the DFT/6-311G(d,p) and lanl2dz levels. The theoretical calculations demonstrate that Pathway I is a more dominant route than Pathway II; therefore, the hydrolysis takes place before complex formation. The DFT/B3LYP theory has been successfully employed using the 6-311++G(d,p) basis set for nppeieoH and the lanl2dz level for [Pd(nppeieo)(ninap)] to support the experimental findings and to evaluate some important parameters, bond length, bond angle, frequency, Mulliken charge distribution, HOMO-LUMO energy gap (ΔE), molecular electrostatic potential (MEP), *etc.* In order to study the electronic properties of nppeieoH and its palladium(II) complex, the theoretical calculations were successfully performed by using the TD-DFT method. The calculated data were in agreement with the observed data. The non-linear optical properties were also computed for all of the compounds, and the results showed a good nonlinear optical property.

## Supplementary Materials

CCDC 1056835 and 1056345 for nppeieoH and its palladium(II) complex, contain the supplementary crystallographic data for this paper. These data can be obtained free of charge via http://www.ccdc.cam.ac.uk/conts/retrieving.html (or from the CCDC, 12 Union Road, Cambridge CB2 1EZ, UK; Fax: +44 1223 336033; E-mail: deposit@ccdc.cam.ac.uk). Supplementary materials can be accessed at: http://www.mdpi.com/1420-3049/21/1/52/s1.
