# Supplementary material for: Synthesis, Spectroscopic, Structural and Quantum Chemical Studies of a New Imine Oxime and Its Palladium(II) Complex: Hydrolysis Mechanism
Source: Molecules. 2016 Jan 21;21(1):52. doi: 10.3390/molecules21010052 (PMC6274148; doi:10.3390/molecules21010052)
Supplement: Supplementary file 1 [file molecules-21-00052-s001.pdf]

Supplementary Materials: Synthesis, Spectroscopic, Structural and Quantum Chemical Studies of a New Imine Oxime and Its Palladium(II) Complex: Hydrolysis Mechanism

Yunus Kaya [1,*,†], Veysel T. Yilmaz [1,†] and Orhan Buyukgungor [2,†]

Table S1. Selected bond lengths (Å) and angles (°) for nppeieoH and [Pd(nppeieo)(ninap)].

Bond Type	NppeieoH	Bond Type	[Pd(nppeieo)(ninap)]
	6-311++G(d,p)		Lanl2dz
Bond lengths (Å)			
C7-N3	1.278	C10-N4	1.330
C8-N2	1.277	C9-N3	1.352
N2-O3	1.392	N3-O5	1.282
O3-H3A	0.964	C7-O2	1.312
		C8-N1	1.370
		N1-O1	1.277
		Pd1-N1	2.065
		Pd1-N3	2.032
		Pd1-N4	2.081
		Pd1-O2	2.111
Bond angles (°)			
C8-N2-O3	111.4	C10-N4-C17	122.6
C7-N3-C15	122.1	C9-N3-O5	124.5
		C8-N1-O1	120.5
		N1–Pd1–O2	79.1
		N1–Pd1–N3	100.6
		N3–Pd1–N4	80.3
		N4–Pd1–O2	100.0

Table S2. Crystallographic data and structure refinement for nppeieoH and [Pd(nppeieo)(ninap)].

Identification Code		nppeieoH	[Pd(nppeieo)(ninap)]
Formula		$C_{16}H_{16}N_3O_3{\cdot}1/2H_2O$	$C_{24}H_{19}N_5O_7Pd$
Molecular weight		306.32	595.84
Crystal system		Monoclinic	Monoclinic
Space group		C2/*c*	C2/*c*
Unit cell dimensions			
a (Å)		14.5050(6)	15.7541(7)
b (Å)		7.0700(2)	15.3662(9)
c (Å)		32.2241(13)	25.9927(11)
α (°)		90.000	90.000
β (°)		107.224(3)	100.716(3)
γ (°)		90.000	90.000
Volume $(Å)^3$		3156.4(2)	6182.6(5)
Z		4	8
Calculated density (g/cm^3)		1.285	1.280
F(000)		1280	2400
μ (mm^{-1})		0.090	0.644
Crystal size (mm)		0.61 × 0.35 × 0.08	0.17 × 0.10 × 0.05
Temperature (K)		293(2)	296(2)
Max. and min. transmissions		0.9545; 0.9921	0.9307; 0.9739
Index ranges		−17/17, −8/8, −39/39	−19/19, −19/19, −32/32
θ range (°)		1.32; 26.15	1.87; 26.50
Reflections collected		16106	18425
Independent reflections		2985 [R_{int} = 0.0642]	6403 [R_{int} = 0.1963]
Reflections observed (> 2σ)		2171	2033
Parameters		212	334
Final R indices [I > 2σ(I)]	R_1	0.0506	0.0483
	wR_1	0.1089	0.0831
Goodness-of-fit on F^2		1.043	0.645
Largest diff. peak and hole ($e{\cdot}Å^{-3}$)		−0.284; 0.281	−0.257; 0.348

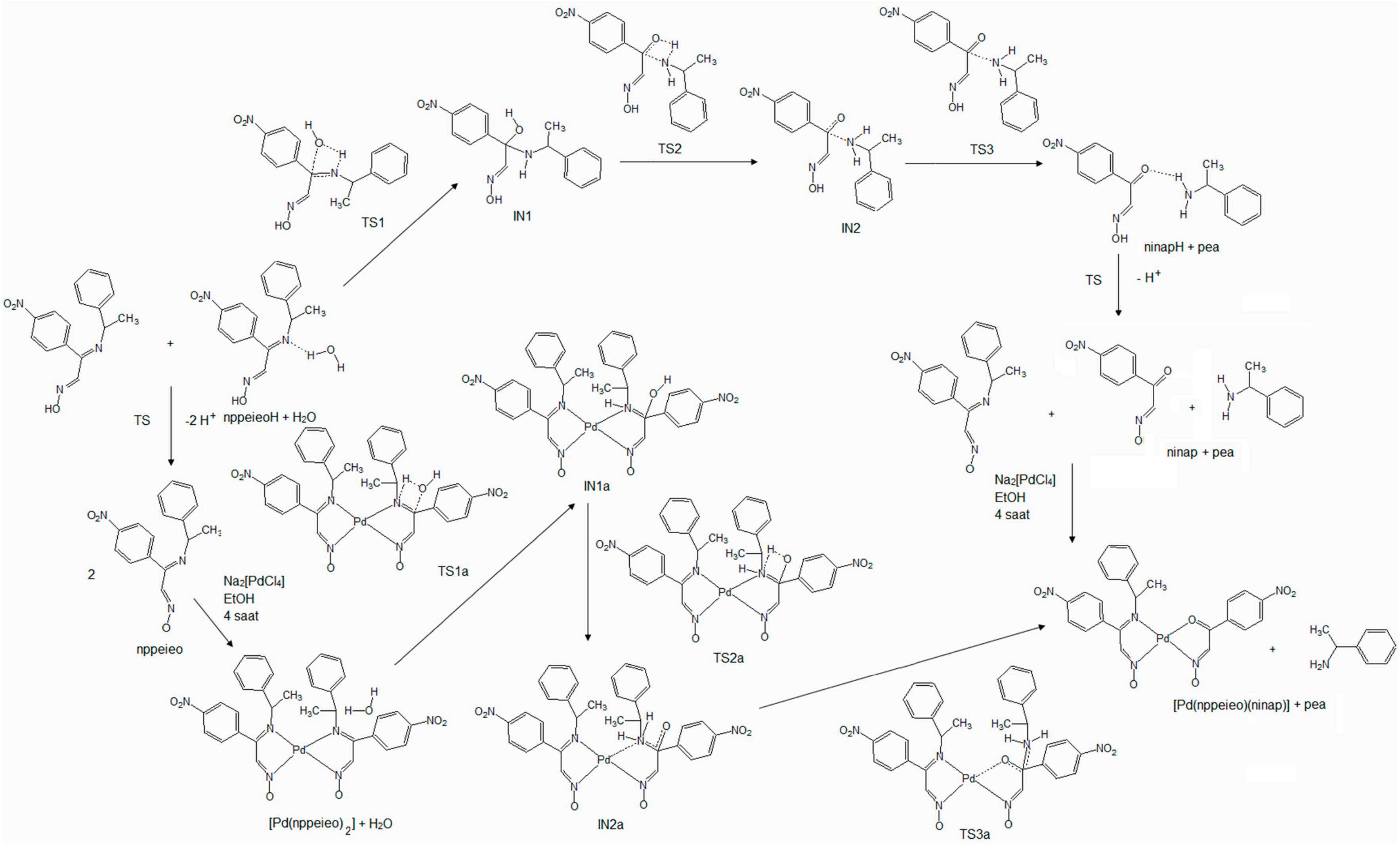

Scheme S1. Two pathway mechanisms of hydrolysis of the imine oxime compound, nppeieoH.

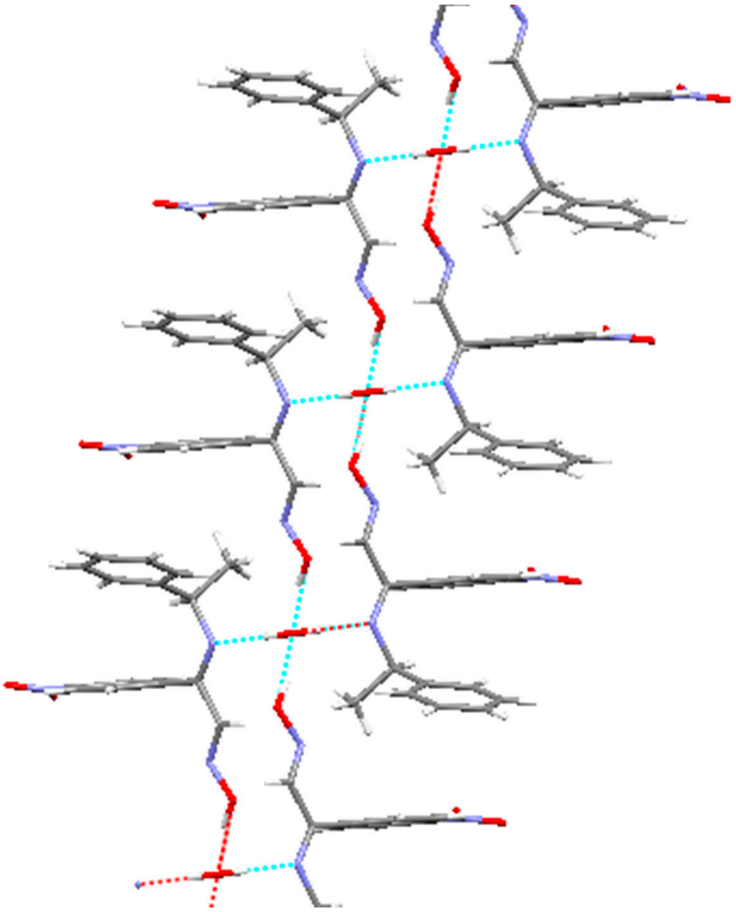

Figure S1. Packing of molecules of nppeieoH viewed down b.

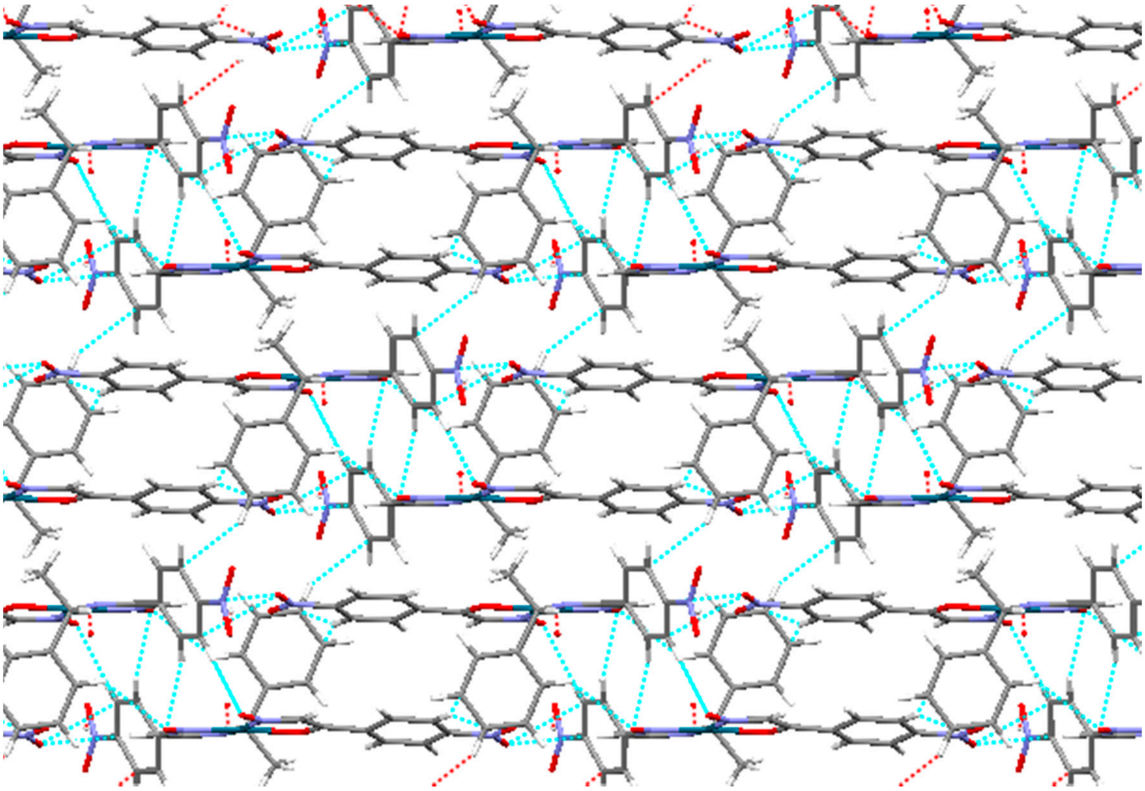

Figure S2. Packing of molecules of [Pd(nppeieo)(ninap)] viewed down c.

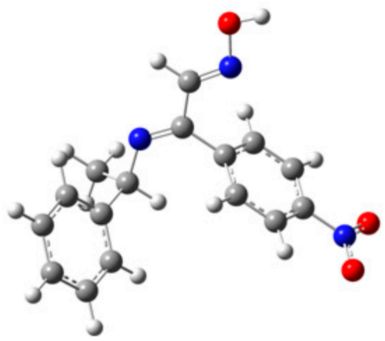

(a)

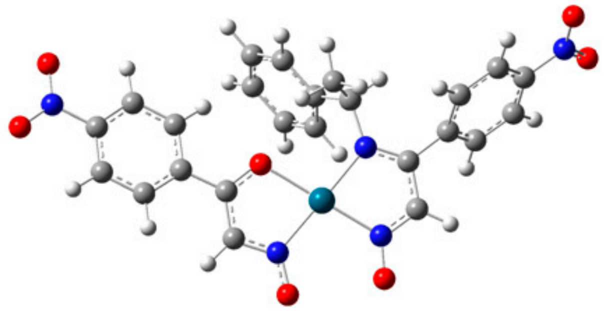

(b)

Figure S3. Optimized structures of: (**a**) nppeieoH; (**b**) [Pd(nppeieo)(ninap)].

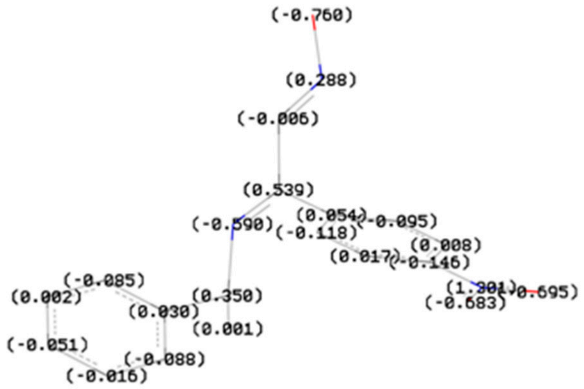

(a)

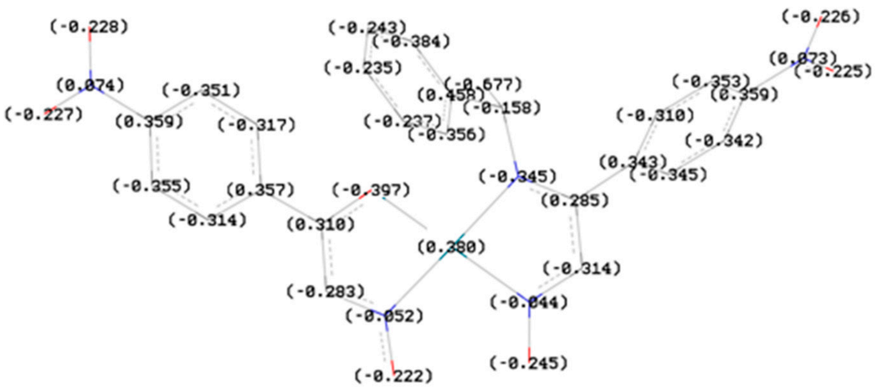

(b)

Figure S4. Mulliken atomic charges of (**a**) nppeieoH and (**b**) [Pd(nppeieo)(ninap)].

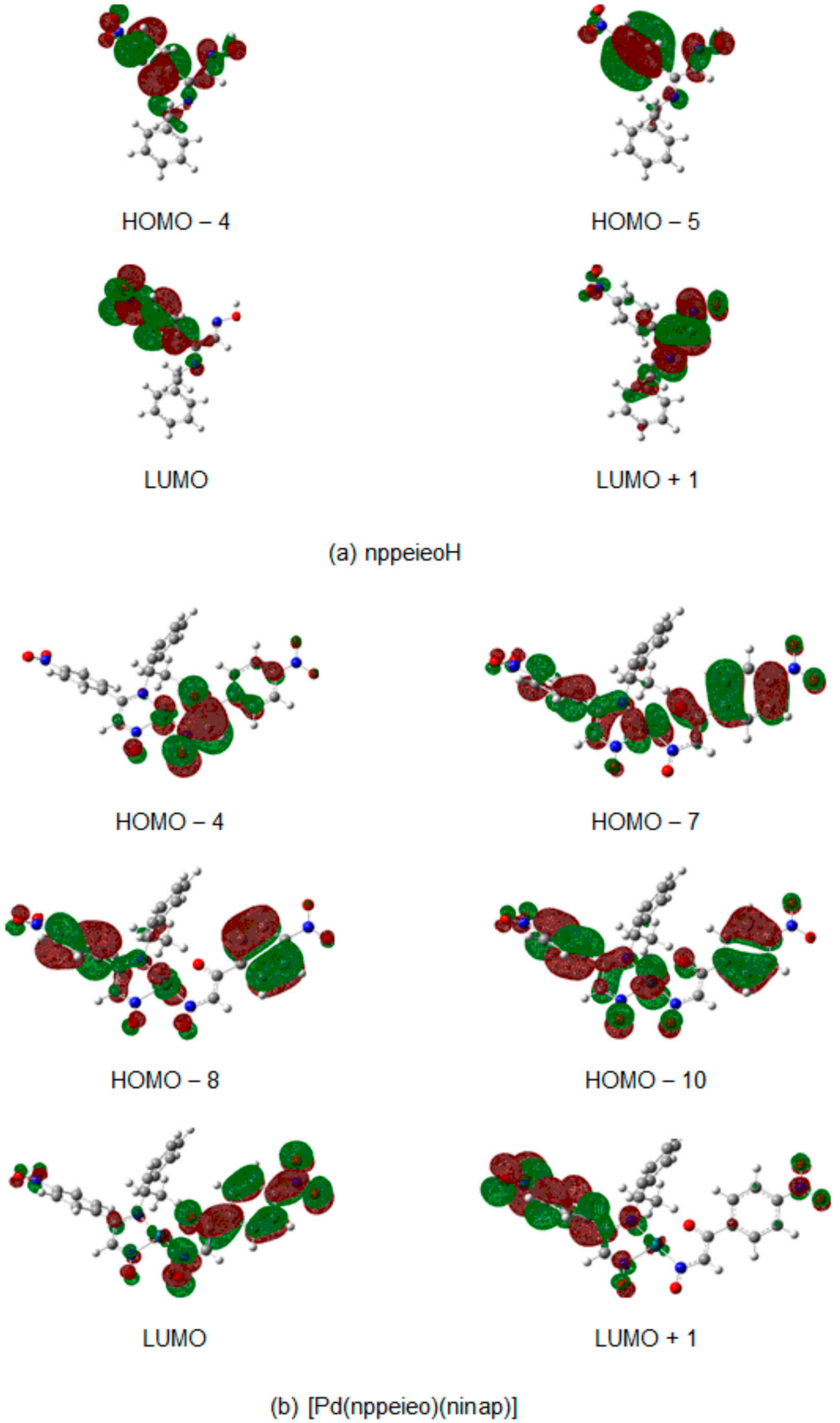

Figure S5. Molecular orbital surfaces for the HOMOs and LUMOs orbitals of: (**a**) nppeieoH; (**b**) [Pd(nppeieo)(ninap)].
